# Community engagement and involvement in Ghana: conversations with community stakeholders to inform surgical research

**DOI:** 10.1186/s40900-021-00270-5

**Published:** 2021-07-05

**Authors:** Karolin Kroese, Bernard Appiah Ofori, Darling Ramatu Abdulai, Mark Monahan, Angela Prah, Stephen Tabiri

**Affiliations:** 1 Global Health Research Unit on Global Surgery, University of Birmingham, Birmingham, UK; 2 Global Health Research Unit on Global Surgery, Ghana Research Hub, University for Development Studies (School of Medicine and Health Sciences) and the Tamale Teaching Hospital, Tamale, Ghana; 3 University of Birmingham, Birmingham, UK

**Keywords:** Patient and public involvement, Community engagement and involvement, Low- and middle- income countries, Ghana, Surgery, Communities, patients, patient contributors

## Abstract

**Background:**

Involving patients and communities with health research in low- and middle-income countries (LMICs) contributes to increasing the likelihood that research is relevant in local context and caters to the needs of the population, including vulnerable and marginalised groups. When done right, it can also support empowerment of wider communities in taking ownership of their own health, lead to increased access and uptake of health services and generally improve the wellbeing of individuals. However, the evidence base of how to undertake successful community engagement and involvement (CEI) activities in LMICs is sparse. This paper aims to add to the available literature and describes how the Global Health Research Unit on Global Surgery’s (GSU) team in Ghana worked collaboratively with the Unit’s team in the UK and a UK-based Public Advisory Group to involve community stakeholders in rural Ghana with surgical research. The aim was to explore ways of reaching out to patients and community leaders in rural Ghana to have conversations that inform the relevance, acceptability, and feasibility of a clinical trial, called TIGER.

**Methods:**

As this kind of larger scale involvement of community stakeholders with research was a novel way of working for the team in Ghana, a reflective approach was taken to outline step-by-step how the GSU team planned and undertook these involvement activities with 31 hernia patients, two Chiefs (community leaders), a community finance officer and a local politician in various locations in Ghana. The barriers that were experienced and the benefits of involving community stakeholders are highlighted with the aim to add to the evidence base of CEI in LMICs.

**Results:**

GSU members from the UK and Ghana planned and organised successful involvement activities that focused on establishing the best way to talk to patients and other community stakeholders about their experiences of living with hernias and undergoing hernia repairs, and their perceptions of the impact of hernias on the wider community. The Ghanaian team suggested 1:1 conversations in easily accessible locations for rural patient contributors, creating a welcoming environment and addressing contributors in their local dialects. A UK-based Public Advisory Group helped in the initial stages of planning these conversations by highlighting potential barriers when approaching rural communities and advising on how to phrase questions around personal experiences. Conversations mainly focused on understanding the needs of hernia patients in rural Ghana to then incorporate these in the design of the TIGER trial to ensure its relevance, acceptability and feasibility. When talking to patient contributors, the GSU teams found that they were more likely to open up when they knew members of the team and the opportunity to speak to local leaders only arose because of the Ghanaian team members being well-respected amongst communities. The experiences of the patient and community contributors led to changes in the study protocol, such as including women in the patient cohort for the trial, and allowed the GSU teams to confirm the relevance and acceptability of this trial. These conversations also taught the team a lot about perceptions of health in rural communities, allowed the Ghanaian team to establish relationships with community leaders that can be utilised when future studies need input from the public, and has changed the minds of the Ghanaian research team about the importance of involving patients with research.

**Conclusion:**

This paper contributes to the evidence base on successful CEI activities in LMICs by providing an example of how CEI can be planned and organised, and the benefits this provides. The conversations the teams had with patient contributors in Ghana are an example of successful patient consultations. Even though there are certain limitations to the extent of these involvement activities, a solid foundation has been built for researchers and community stakeholders to establish relationships for ongoing involvement.

**Supplementary Information:**

The online version contains supplementary material available at 10.1186/s40900-021-00270-5.

## Plain English summary

Evidence in the literature shows that research that was designed and implemented with the help of patients or other members of the community affected by the study, is more likely to be relevant to the needs of the end user. This can have positive effects on the quality and impact of the study. However, working collaboratively with patient or public contributors can have certain challenges, especially in low- and middle- income countries. For example, factors such as different languages or dialects, religious beliefs, health beliefs, level of literacy, understanding of research, and poverty can potentially make it more difficult for researchers to reach, communicate and involve relevant members of the public. On top of this, the evidence base for successfully implementing these so-called community engagement and involvement activities is sparse, making it more challenging for researchers to learn from others’ experiences.

Members of the Global Surgery Unit, who are based in Ghana, proposed a clinical trial to address the shortfall of specialist surgeons at rural hospitals. This will be achieved by training medically qualified doctors, who have not yet gone through the specialist training to become qualified surgeons, to perform inguinal hernia repairs proficiently. Before the study gets funded and a protocol can be designed, the researchers need to ensure its relevance, acceptability, and feasibility. This is usually done by getting the opinion and thoughts of key stakeholders, mainly individuals who will be affected by the research. To achieve this, the team in Ghana worked together with Global Surgery Unit members in the UK to identify the best ways to approach and have conversations with 31 inguinal hernia patients who either previously had surgery or currently live with hernias, two Chiefs (community leaders), a community finance officer and a local politician in a variety of locations in Ghana that are representative of the patient cohort for the study. The intention was to understand the impact of hernias on individual patients, their families, and entire communities to inform the relevance, acceptability and feasibility of the clinical trial, and ensure that relevant aspects are captured in the study protocol. A UK-based public advisory group provided initial input to help eliminate potential barriers of addressing patient contributors in rural Ghana.

This paper states in detail how these conversations were planned and organised, the challenges the team had to overcome, and what they learned and gained from talking to community members - which goes far beyond just informing a study.

**Supplementary Information:**

The online version contains supplementary material available at 10.1186/s40900-021-00270-5.

## Background

Patient and Public Involvement (PPI), or Community Engagement and Involvement (CEI) as often referred to in global health research, is a major contributor to increasing the likelihood of research being relevant to and benefitting the population affected by it. The best way to ensure this is by involving relevant community members at all stages throughout the research cycle – from designing the study to its implementation and dissemination of findings [1]. While the definition of and even the terminology used for CEI can vary, its essence is captured by the US Centres for Disease Control and Prevention when defining community engagement: “… the process of working collaboratively with and through groups of people affiliated by geographic proximity, special interest, or similar situations to address issues affecting the well-being of those people” [2]. In health research, involving patients and the public has been shown to increase the relevance and value of the study by identifying the needs of the affected patient group or wider community, inform the feasibility and accessibility of the study intervention by identifying barriers to participation, and enhance recruitment by raising awareness of the intervention and its benefits. Research that is relevant and accessible to patients means that funding is not wasted on something that the end-user cannot or does not want to use and increases the success rate of the study and the likelihood for future funding [1, 3]. This is why most funding bodies have now made it a requirement to incorporate a robust plan for CEI in all grant applications. This becomes even more relevant in lower resource settings, where research should aim to cater for the needs of the most marginalised, but inequalities due to language, understanding of research and poverty mean the gap between the research world and the local population is even bigger than in high-income countries [4].

Thus, the rationale for involving communities with research in low- and middle- income countries (LMICs) is not limited to solely ensuring the success of a research project. It is about providing the tools to involve the local population in all aspects of decision-making and implementation of research, with local ownership improving transparency and accountability of research and research funding. This can then lead to optimal resource allocation across diverse settings that builds on local capacity and potentially leads to research findings being accessible to and utilised by the ones in need [5].

With most funding bodies sitting in the Global North, it is crucial to ensure that research prioritisation and implementation in LMICs is led on by the Global South – here specifically representing the needs of people at grassroots level. Only then can research become meaningful and sustainable [5].

The National Institute for Health Research (NIHR)- funded Global Health Research Unit on Global Surgery (GSU) at the University of Birmingham has established a network of research hubs in LMICs to conduct surgical research. Each hub is led by a local surgeon and their team of clinical, as well as non-clinical staff. Surgical research across all hubs in form of clinical trials is prioritised annually by GSU researchers from all participating LMICs, as well as the UK-based team. Furthermore, smaller-scale pump priming studies or single site clinical trials, which can establish a proof of concept to develop larger multi-site or multi-country trials, are also funded. These are country-specific, addressing urgent health needs in individual LMICs.

In sub-Saharan Africa, a major health concern is inguinal hernias, reported to affect 10% of the overall population and up to 37% of men over the age of 55 [6]. The limited surgical workforce and the cost implications for patients traveling to hospitals and undergoing surgery are believed to pose a serious threat to entire communities. The research hub team in Ghana therefore proposed a clinical trial focusing on task-shifting in rural hospitals to build on local capacity by training non-surgeon physicians to specifically perform inguinal hernia repairs. Hernia repairs are usually do not require complex technique. The Ghanaian team called this trial TIGER (Task shifting inguinal hernia repair). The aim is to reduce the burden of hernias on communities by increasing the effective surgical workforce. Task-shifting is the delegation of specific specialist tasks to less qualified health workers and has been shown to increase surgery volume. However, careful consideration is needed about the relative risk of postoperative surgical outcomes between operations performed following task-shifting versus surgeons [7]. Training non-surgeon physicians in one specific surgery over the course of a weekend with supervision by experienced surgeons for a further few months allows for faster turnaround of a niche, skilled workforce rather than waiting years for surgeons to finish their training and reach full proficiency across the board.

Working closely with the local team in Ghana based at the University for Development Studies (School of Medicine and Health Sciences) in Tamale and the Tamale Teaching Hospital, the Unit’s UK-based CEI Manager set out to explore ways to reach out to patients with lived experience and community leaders in rural Ghana to understand the extent of the burden hernias pose on patients and whole communities. Listening to patient stories and understanding the experiences of people affected by hernias prior to designing the study protocol allowed the team to assess the relevance of TIGER, tailor the trial to local context, and ensure that research funding was allocated to areas of need.

With there still being a shortfall of evidence and inconsistency in recording and reporting of community involvement with global health research studies [4], this paper aims to provide step-by-step information on how successful involvement activities can be designed in similar settings. The paper does not only outline the reasons for involving community stakeholders and a potential way of implementing this; it also focuses on the barriers to high-quality involvement of community stakeholders in low-resource settings and limitations of the described activities for shared learning across the global health research network.

## Methods

This paper outlines how community engagement and involvement activities were designed and implemented in Ghana in September 2019 and highlights the challenges and solutions as perceived by the GSU teams based in the UK and Ghana.

To explore the relevance and feasibility of the TIGER trial in rural areas of Ghana, the UK-based CEI Manager, Dr. Karolin Kroese, worked closely with the local research team, specifically the Research Hub Lead Prof Stephen Tabiri, Hub Manager Bernard Ofori Appiah and Research Nurse Darling Ramatu Abdulai. Karolin provided expertise on best practice CEI in line with the UNICEF standards (5, at draft stage at the time) from a high-income country angle, which was then adapted to local context by the Ghanaian team and a UK-based public advisory group (PAG), as outlined in the results section.

Preliminary advice on CEI activities and Ghanaian culture was provided by the PAG, specifically the Ghanaian member Angela Prah. The group consists of six members who live in the UK but are originally from some of GSU’s collaborating LMICs: Ghana, Nigeria, The Philippines, and India. The group presents an opportunity to provide initial advice on potential barriers, challenges and solutions when undertaking research or community engagement and involvement activities in LMICs. All members of the group were reimbursed for their time according to the NIHR National Standards for Public Involvement [8]. The CEI activity was reported and evaluated using the GRIPP-2 framework ([9], Additional file 2: Appendix 3).

This paper was written in collaboration with the Ghanaian team and the public contributor, Angela Prah. Patients and community representatives in Ghana were not involved in writing this paper due to language barriers. However, when talking to patients and community leaders in regard to TIGER, consent was given for their views and opinions to be outlined in this article.

### Authors’ statement on ethics and consent

The activities outlined in this publication classify as community engagement and involvement (CEI) with research, as opposed to being research. Therefore, the team did not need ethics approval according to the NIHR Involve guidelines. Conversations with patient and community contributors did not form part of a qualitative study, and therefore, no research methodology was applied to analyse findings or extract data from the conversations. Patient contributors shared their lived experiences, allowing the team to understand what mattered to this (small, yet representative) group of individuals when undergoing treatment for their hernias. This insight allowed GSU teams to gauge how relevant the proposed TIGER study is, who the patient cohort might be, and if the suggested protocol can feasibly be implemented in Ghana. Input from contributors led to recommendations being outlined for the TIGER researchers, who as a result decided to implement these when designing the study protocol. The patient contributors whose stories form part of this article are a different group to the future participants of the TIGER trial. Ethics for the trial will be obtained as per guidelines when the protocol has been finalised based on feedback from the patient contributors.

The team decided to ask contributors to sign consent forms at the start of the conversations. This was suggested by the local team with the main concern being around language barrier and the worry about community members potentially confusing research and CEI. The team wanted to be certain that contributors knew what was asked of them and equally understood the intentions of the team, not least to manage expectations. Contributors were given the opportunity to study the consent form, have the different points read out and explained to them, and then consent to the points they felt comfortable with. Some contributors did not consent to their pictures being taken and uploaded on GSU webpages, but all indicated they understood the team’s reasons for the conversation, felt comfortable sharing their stories, knew they did not have to talk about any experiences they did not want to share, and agreed to the conversations being audio-recorded. This last point was relevant for the UK-based team to ensure communication between patient contributor and translator could be followed.

## Results

### Designing and planning of CEI activities for TIGER: involving the public in involving the public

TIGER was proposed by the Ghanaian research team as a study to potentially reduce the risks of surgical complications and increase access to safe surgery in rural parts of Ghana, therefore specifically supporting the health and wellbeing of a very vulnerable population of this country. Before funding from GSU could be released, the burden of hernias specifically on rural communities and therefore the relevance of this study, as well as its feasibility and likelihood of uptake by patients had to be explored. The UK-based CEI Manager therefore proposed consulting hernia patients with lived experience in rural Ghana, as they are representative of the patient cohort for TIGER.

To shape the CEI activities, a meeting with the UK-based PAG was hosted at the University of Birmingham to discuss where and how community stakeholders in Ghana can help inform the TIGER study. Particular focus was laid on barriers to involvement and potential solutions. The PAG advised to explore how patients felt about the study intervention, specifically about non-surgeon physicians performing surgeries. When looking into the proposed TIGER study design, the group furthermore highlighted barriers for patients to attend hospital appointments and participate in clinical trials, such as travel expenses, including an additional trip for follow-up for study participants. It was decided to explore this further in conversations with patient contributors in Ghana.

Based on the discussion with the PAG, the GSU teams decided to draft a template for guided discussions (Additional file 1: Appendix 1 and 2) with patient and community contributors in Ghana, with the overarching aim for patients to share their personal experiences, which will allow the team to address the following areas:

1. Access to hernia surgery – capturing patients’ stories on seeking help for their hernias.
  Ultimately assessing the relevance of the TIGER trial in rural communities.
2. Acceptability of the intervention to patients – Would patients be comfortable with a trained non-surgeon physician, rather than a surgeon, performing their surgery? Focusing on what matters to patients in rural Ghana when undergoing hernia repair.
3. Feasibility of delivering the trial intervention with a focus on access to care and in-person follow-up – Will additional travel back to hospital for follow-up cause any problems for study participants?

The next step was to develop a detailed action plan. Over several weeks, the main mode of communication between the UK and Ghanaian teams was via WhatsApp. This group chat proved to be the most successful way to hold each other accountable and ensure the project progressed in a timely manner. The occasional teleconference was used for more in-depth discussions and updating the wider team.

In this time, leading up to a 7-day visit of the UK-based team (Table 1), travel arrangements were taken care of by the Ghanaian team, including the itinerary of hospital visits to talk to the patient contributor, booking hotels and arranging meetings with community Chiefs and religious leaders in rural villages. For safety, access, and translation reasons, it was decided that the local team would accompany the UK team on their hospital visits.

**Table 1:** Itinerary of the Ghana road trip

Day 1	Arrival in Accra from the UK
Day 2	Accra to Kumasi by plane
	Kumasi to Offinso by car
	Conversations with patient contributors at St Patrick Hospital, Offinso
	Offinso to Nkoranza by car
	Conversations with patient contributors at St Theresa Hospital, Nkoranza
Day 3	Techiman to Wa, Nadowli by car
	Conversations with patient contributors at Nadowli District Hospital
Day 4	Nadowli to Tamale by car
Day 5	Conversations with patient contributors at Savelugu District Hospital near Tamale
Day 6	Tamale to Sunyani, Bono Region by bus
	Meeting Chiefs and community leaders
	Sunyani to Kumasi by bus
Day 7	Kumasi to Accra by plane

The GSU team travelled to various hospitals and villages in rural in Ghana to talk to patient and community contributors

The CEI Manager travelled to Ghana with GSU’s Health Economist, Mark Monahan, who was undertaking a related project looking at the cost implication and feasibility of upscaling hernia repairs at rural hospitals.

### Identifying patient contributors – hospital hopping

Four mainly rural hospitals that work with Prof Tabiri and the Tamale Teaching Hospital, and will be recruiting patients to the TIGER trial, were chosen to be the most representative for the patient cohort (Table 2) and therefore allow the teams to speak with relevant community members with lived experience of living with hernias and undergoing hernia repairs at the local hospitals.

**Table 2:** Location of hospitals

Hospital	Region	Interviewed patients
St Patrick Hospital	Offinso, Ashanti Region, near Kumasi	8 Patients (4 pre, 4 post surgery)
St Teresa Hospital	Nkoranza, Bono East Region	10 patients (5 pre, 5 post surgery)
Nadowli District Hospital	Nadowli, Upper West Region	8 patients (6 pre, 2 post surgery)
Savelugu District Hospital	Savelugu, near Tamale in the Northern Region	5 patients (2 pre, 3 post surgery)

The table provides the names and regions of the hospitals and the number of patients that the team talked to. Patients at four rural hospitals closely working with the GSU Research Hub at Tamale Teaching Hospital were approached and meetings with community leaders took place in Odumase, the Bono Region of Ghana

Whilst planning the CEI activities, the Ghanaian team contacted the hospitals to ensure that the staff were happy to identify patient contributors from either previous or current hernia patients and provide an office or hospital room where private conversations could take place. It proved immensely beneficial that the local team was well- connected and –respected, with hospital staff immediately agreeing to support the activities. Hospital staff then announced Prof Tabiri’s visit via local radio stations a few days prior to arrival. On the day, patient contributors voluntarily travelled the short distance to their local hospitals in order to take part in the conversations, not least because many of them were treated by Prof Tabiri previously and trusted him and his team.

To represent the patient cohort eligible to participate in the TIGER trial, community members 18 years and older could take part in the conversation with no restriction on male and female numbers. Overall, the team talked with 17 patients contributors pre- and 14 patients contributors post- hernia surgery, with six out of the 31 patients contributors being female (Table 2). Patient contributors and community leaders were each reimbursed for their time and travel costs with 50 Ghanaian cedis, which was suggested by the Ghanaian team as an appropriate amount. Patients contributors pre- surgery were also provided with a flat mesh, a sterilized polypropylene mesh commonly used in lower-resource settings (7x15cm), for their hernia repair, which they usually have to pay for themselves.

### Conversations with patient contributors

Because people in rural communities speak multiple dialects and very little or no English, the Ghanaian team suggested that 1:1 conversations were the best way to communicate. The main concern was around patient contributors potentially misunderstanding the reasons for being approached or confusing the conversations as part of involvement activities with participating in research. The team therefore ensured that a translator was present for each conversation to directly translate between the Ghanaian dialect and English as spoken by the GSU team members. Each conversation started with the team outlining the reasons for approaching the patient contributor and highlighting the difference between this involvement activity and research. Only when the team was confident that the contributor fully understood the nature of the conversation and was happy to proceed, the directed conversation around their lived experiences started. The team even provided the contributors with forms to indicate their understanding of the team’s intentions, and their consent to be quoted in any kind of publication, or their pictures being taken for GSU webpages. Patients contributors consented by either signature or thumbprint and conversations were audio-recorded for analysis. When the translator was not part of GSU or the Ghanaian team, but a member of staff at the local hospital or a patients’ relative, they were reimbursed for their time and travel costs with 50 cedis. When patient contributors’ relatives acted as translators, the team experienced some difficulty with the quality of the translation due to them interpreting the patient’s answers and providing a summary in English rather than a word-for-word translation. The members of the Ghanaian team ensured that the patients contributors felt comfortable throughout the interview. This proved immensely important for the quality of the interaction. They visibly relaxed when they identified members of the Ghanaian team, who they were either familiar with due to previous treatment at their hospital or had heard of before.

The conversation was loosely structured around the focus areas as identified by the PAG and outlined above: To capture the patient contributors’ lived experiences to inform relevance, acceptability, and feasibility of the TIGER intervention. A script for this was written based on whether the contributors were previous hernia patients or currently living with hernias (Additional file 1: Appendix 1 and 2). During the conversation, the team ensured to allow the contributors to provide as much detail about their experiences as they wanted and felt comfortable with, and only asked directed questions when necessary to address the three areas of concern.

### Patient contributors’ feedback: lived experiences summarised

Summarised below are the overall topics raised by the patient contributors, as well as opinions from other relevant community members, not only addressing the three areas of concern as outlined above (study relevance, feasibility and acceptability), but providing the team with further insights into challenges and barriers of seeking healthcare and needs of rural communities. All information below stem from these conversations and represent the views and experiences of the patient contributors. It is important to note again that these conversations were not qualitative research. T herefore, no methodology was used to analyse themes. The GSU CEI Manager and Ghanaian team merely summarised common opinions and experiences as stated by the patient contributors und used these to inform the TIGER trial with respect to protocol design and trial intervention.

### Relevance of study

#### Surgical capacity in rural areas is urgently needed

Most rural hospitals do not have a surgeon on site at all times. Sometimes the surgeon only visits once a week or less, as they have to cover multiple hospitals in the area. Their arrival at the hospital will be announced via the local radio, information centres, churches and mosques, with patients lining up in large numbers outside the hospital and surgeons performing all kinds of surgery, no matter their specialty. More often than not, patients are being sent home or allocated a bed or any kind of free space in the hospital for several days without having had surgery, as the medical team just does not have enough capacity. This means that patients will have to either travel to the hospital multiple times whilst being ill, or that they have to stay in the hospital for several days, often sleeping on benches or the floor. This has severe consequences on the patient and their families.

Most people in rural Ghana are farmers or tradesmen, selling goods along roads to be able to feed their families. Taking time ‘off’ means that they cannot tend to their fields and most likely don’t only lose out on goods to sell, but also on harvesting and providing food for their own meals, meaning more expenditure having to buy in supplies. On top of that, patients and their family members traveling with them have to spend money they do not have on transport to and from the hospital additional to the cost of the surgery. Most patients will travel by bus, only a few with severe pain ask friends or family members with motorbikes or cars to take them. According to a Chief the team spoke to, as well as a financial officer, occasionally community leaders cover these costs when families cannot manage to save up the needed funds themselves.

#### Barriers to accessing healthcare: traditional medicine and alcohol are considered feasible treatment options amongst patients– whereas surgery is considered unsafe

Many communities in rural Ghana are under the impression that traditional medicine or alcohol can cure inguinal hernias or at least keep the pain at bay enough to fully function and be able to continue with their everyday life and work. Patients often live for years with a hernia - most pre-surgery patients that the team talked to have lived with their hernia for 5–10 years prior to seeing a doctor due to worrying about the cost implication, as well as potential poor outcomes of surgery. Stories about patients dying after surgery are well spread in rural communities. A more affordable, and in their opinion, safer option is traditional medicine and alcohol, even though these are arguably not cheaper than surgery when taken over many years – and only treat the symptoms, not the underlying cause.

#### Community leaders confirm, lack of access of safe hernia repairs poses financial burden on wider communities

After having heard about community leaders covering costs of surgery for patients within their district, the local team made arrangements to separately meet two Chiefs, a Catholic Priest, an Islamic Cleric, and an Assemblyman in the Sunyani West District in the Bono Region. Discussions with these well respected community leaders confirmed the impact that poor access to safe, affordable surgery has in these rural areas. The team was invited to meet with the Chiefs and their community elders to raise awareness of TIGER and explore their opinion on its relevance. Usually, anyone enquiring about a meeting with the Chief would be asked to follow strict rules of paying respect. However, GSU researchers were allowed to address the Chiefs directly with the help of a translator, which demonstrated the immense interest these community leaders had in finding out more about the benefits of this research for their communities. The conversations highlighted that community leaders will often use money meant to support community projects, such as fixing roads and furthering general infrastructure, for patients to be able to undergo surgery and help support their families. After explaining TIGER to them, all community leaders stated that this kind of task-shifting around hernia repairs, as well as other surgeries, would relieve a substantial amount of this burden on their communities. It was also highlighted that there is stigma attached to hernias in rural communities, with people not understanding the reasons for this condition and the impact it may have on their overall health and wellbeing. It is therefore important that the study results are shared widely in an accessible way. The relationship with these community leaders will support this dissemination and will ensure that future research can be informed by representative community members.

### Acceptability of intervention: patients trust medical staff and have faith in god

When it came to addressing the matter of surgeons versus non-surgeon physicians performing the hernia repair, it became clear early on that patients will trust medical professionals and their opinion. If a surgeon vouches for a non-surgeon to have been adequately trained, the patient will not question this. In fact, most post-hernia surgery patients contributors stated that by the time they finally sought help and made their way to the hospital, they did not care who fixed their hernia, as the pain was unbearable. When prompted if they would object to someone who has not had years of surgical training undertaking their surgery, their response often was that God would guide the surgeon’s hand and that they have faith.

### Feasibility of study intervention

#### Participating in clinical trials for the greater good of the community

When addressing having to travel to the hospital for follow-up as a participant in the TIGER trial, it was argued that if patients travel to hospital and will be seen by a surgeon right there and then, cost implication on their families will be minimised. A second trip to the hospital for follow-up after surgery will therefore still have less impact on the family’s financial status than the current situation of having to travel multiple times or stay at the hospital for a prolonged period.

#### Adaptation of study protocol: inclusion of women in patient cohort

Changes to the trial protocol were made as a result of the conversations with patient contributors. For instance, initially, women were excluded from the study, but lived experiences of female patient contributors convinced the local team to change this. It was furthermore established that dissemination of relevant, accessible information about surgery and living with hernias will be crucial to ensure that communities experience the benefits of this study. The experiences in Ghana have opened up discussions at GSU about whether educational interventions may be needed to support better health in general so that the population can be provided with the knowledge and tools to help themselves.

### Reflections of the research team

Traveling around Ghana and talking to patient contributors has not only informed the TIGER trial, but has made the GSU teams aware of the impact CEI may have generally on shaping and informing clinical research. After initially doubting the benefits of talking to communities that have never even heard of clinical trials, health research or CEI before, these involvement activities completely changed Prof Stephen Tabiri’s and Bernard Ofori Appiah’s mind about how they will design studies in the future.

In conversation with Bernard a few weeks after the CEI activities, he said:” Imagine an architect being [hired to design] someone’s house and then the architect […] will not get back to the person to find out what the needs of the person are and [does not] check the topography of the land and then he designs the house. At the end, the person will not be happy.

Or a tailor. You consult a tailor for a dress for a wedding. The tailor will not measure you out or interview you to find the colours you want and then he sews something for you. I believe at the end of it all, you might not be happy.

The same way research should go. At the end of it all, the benefit should be for research [and its] community focus.

So why do we design research without consulting [patients]?”

Angela Prah is a member of the UK-based PAG, having moved to the UK from Ghana to undertake a Master’s degree at the University of Birmingham. Her background is in healthcare in rural Ghana, where she worked closely as a nurse with patients and local communities. Angela’s expertise in working with local communities, having grown up in rural Ghana, was invaluable in shaping the CEI activities. Her thoughts on the TIGER trial, the relevance of working with patients in LMICs, and her feedback on what she gained from being involved with GSU are summarised below.

“Involving local communities - this being patients, their community and their caregivers - is an integral part of healthcare, however, as much as it is important in achieving optimum health, it is less considered in LMICs. The confidence a patient has in accepting to undertake a procedure, in this case a surgical procedure - be it minor or major - depends on their understanding of the whole process from pre, intra and post procedure. Being involved gives them more trust in accepting the trial intervention. TIGER trial has been a tremendous help as it would improve the use of patient-family centred approach in delivering a high quality healthcare system. This would in turn increase patient confidence in accepting procedures that would have usually been refused or delayed till they become severe.

Having the opportunity to be part of the advisory group has made an important mark in my career as a caregiver. That is, the knowledge acquired and the skills generated through the TIGER trial has enhanced my abilities to care for patients, involve them in their care and bring their community on board in the healthcare system as the primary concern of healthcare is the patient and their environment (community). This would have a transferrable impact on other healthcare givers as well. I would like to thank the TIGER trial team for choosing Ghana to be a part of the study as this would go a long way to affect positively the healthcare of Ghana, our citizens and our communities. This would inform healthcare practice in the sense that there would be a change in the way and manner we take our patients through procedures by involving them in the process.”

### Summary of implications of the CEI activities

The interaction with patients in Ghana has confirmed that task-shifting and capacity-building research is crucial to support the surgical workforce in rural Ghana, has led to amendment of the TIGER study protocol and supported the research ethics application. It has furthermore allowed the team to get a glimpse at how people in certain areas of Ghana live, perceive health and disease, and their beliefs, struggles and concerns when it comes to health and seeking care. The Ghanaian team see value in involving patients and communities in future studies and have been provided with the tools to lead on this themselves. Lasting relationships with community stakeholders were established (Chiefs, politicians and religious leaders) that can be utilised for future research prioritisation, involvement throughout the research cycle, and dissemination of findings. This is providing a first step towards research being informed, led on and implemented with the help of community representatives in the Global South. A further outcome is that the experiences in Ghana helped implement other high-quality CEI activities in some of the Unit’s collaborating LMICs, with an opportunity to build on the lessons learned and share knowledge and expertise with local teams across the world. Table 3 outlines the top tips for CEI undertaken in Ghana and other LMICs from the UK-based researcher’s perspective.

**Table 3:** Tips for designing CEI activities in LMICs when based in the UK

Tips for CEI in Ghana (or other LMICs) when based in the UK (or other HICs)	
General	• Work closely with a local team (if based in a high-income country)
	• The better connected the local team is within their community, the easier it will be to access and establish relationships with relevant stakeholders, especially high-ranking community members
	• The local team has to have capacity to lead on the CEI activities their end - it is helpful to identify one main point of contact in the collaborating LMIC research team
	• Find out how much the local team knows about CEI and provide training, if needed
	• Be aware that UK (or other guidelines) best practice involvement might not work in local context, remain flexible and let the local team lead on adapting the CEI activities
Planning stage	• Start brainstorming ideas and explore quality CEI activities early on in the research cycle with plenty of time to adapt and re-design
	• Aim for the highest level of involvement possible (community-led, co-produced) and adjust from there according to feasibility and resources
	• Be flexible and prepared to adapt - timelines/approaches will change not only at planning stage, but also when undertaking the activities - A rough guideline to start with might be sufficient to get the ball rolling
	• Keep communication up with the local team when planning the activities, using pathways that suit everyone (WhatsApp, e mail, phone calls, Google docs)
	• Payment of involved community members depends on local standards: Consult local team for appropriate rates
	• An easily accessible advisory group with representative members (e.g. in your country/language) is of benefit when exploring initial steps of CEI planning in LMICs, e.g. informing on barriers, challenges, potential solutions
Undertaking of CEI activities	• Embrace the local culture and be aware of cultural differences – the local team can inform on this
	• Travel with a local person/team (ideally someone well-respected in the communities you visit for better access to community groups, as well as potential meetings with community leaders)
	• A good translator is crucial when aiming to have in depth conversations with community contributors in different languages or dialects
	• Patients will feel more comfortable in conversations when they recognise someone from the research team (Previous relationship-building)
	• Communicate your intentions and reasons for the visit clearly and provide context and background as appropriate to community contributors before undertaking any kind of discussion/focus group/general CEI activity. This will help the contributors feeling safe and understanding what is expected of them, but will also manage their expectations of what you will deliver on as a result of the activity
	• Explore ways to build sustainable, lasting relationships with the engaged patients and communities – let them inform dissemination pathways and ways to keep in touch
After the CEI activities	• De-brief with the local team and explore ways to improve CEI in the future, if needed, with the aim for the local team to independently lead on future activities and incorporate CEI more widely
	• Report the CEI activities (e.g. using the GRIPP-2 framework, shared learning platforms) and implement patient suggestions where possible in your study

This table provides a summary of the top tips from the UK team’s perspective when designing new CEI activities in collaboration with an LMIC-based team

*HIC* High-income country, *LMIC* Low-and middle-income country, *CEI* Community engagement and involvement

## Discussion

### Implementing community contributors’ feedback

The evidence base behind the TIGER proposal was solid, with other task-shifting studies having proven successful in LMICs with focus on improving health system efficiency [10]. WHO describes task-shifting as a ‘viable solution for improving health care coverage by making more efficient use of the human resources already available and by quickly increasing capacity’ [11]. The CEI activities designed for TIGER therefore aimed to confirm the relevance, feasibility and acceptability of this specific intervention focusing on hernia repairs. These are reported benefits of CEI - or PPI in the UK - with clinical trials [3]. Talking to patient contributors and community leaders in various different locations in Ghana, representing the relevant patient cohort for TIGER, has confirmed relevance, feasibility and acceptability of the study, and led to protocol changes to further increase relevance. It has furthermore highlighted the urgent need for this intervention to lessen the burden on patients, families and whole communities.

### Level of involvement of community stakeholders

Designing and implementing CEI activities was a new concept for the team in Ghana. Likewise, exploring feasible ways of involving patients in an LMIC-setting was new for the UK-based team, as well. With still little evidence in the literature, especially on involving community contributors throughout the research cycle and establishing lasting relationships [4], the team set out to adapt what is accepted as best practice in the UK for local context. For this, the team used the UNICEF core standards (which were at draft stage at the time): 1. Participation, 2. Empowerment and ownership, 3. Inclusion, 4. Two-way Communication, 5. Adaptability and Localization, and 6. Building on Local Capacity [5]. CEI with research designed using these standards as a guideline is meant to contribute to the empowerment of communities to take ownership of their health by actively being involved in the research process. For this to happen, equal opportunities have to be created, ensuring that relevant stakeholders in the community get the chance and are provided with the tools to feed into aspects of the research cycle, especially marginalised communities [5]. This may mean that researchers or CEI professionals have to spend time supporting community contributors by, e.g. providing training, or where contributors live in remote locations, travelling to them to perform involvement activities, or designing activities in local language with a translator [8]. These limitations were addressed by the GSU teams, highlighting the relevance for establishing trust and good relationships with patient contributors, and the need for a skilled translator in doing so. It is important to note that these adaptations may vary in different local contexts and the way certain communities can be reached and get involved with research may depend on their skills and resources [5].

The GSU team used the GRIPP-2 framework [9] to highlight the limitations experienced when designing and undertaking the involvement activities in Ghana. Aiming to involve patients throughout the research cycle, which can reportedly have big impact on the quality and success of the study [12], the Ghanaian team was concerned about rural communities potentially not understanding the concept of research or what it means to be involved with research. This concern is often raised by GSU LMIC-based researchers, not only in Ghana. The team’s activities based on conversations with individual patient and community representatives only classify as community consultation, an entry-level involvement activity [13]. However, opening up the conversation about TIGER and exploring the patient journey has allowed the researchers to gauge how interested patients and community leaders are in ongoing involvement with GSU research, gain their trust – a step that has been reported as crucial for meaningful and sustainable engagement and involvement by other researchers, as well [14, 15] - and establish ongoing relationships with community leaders. The Ghanaian team can now utilise these relationships for future involvement activities, potentially even aiming for involvement in research prioritisation.

## Conclusion

This work contributes to an evidence base for CEI activities in LMICs, highlighting the process of designing these activities in collaboration with LMIC-based researchers and UK-based public contributors. This paper outlines what the GSU teams has learned and gained from talking to community stakeholders in Ghana. It also highlights the challenges and barriers, as well as solutions when involving patients in rural areas of Ghana in informing and shaping a clinical trial, with the aim to share these experiences with the wider global health research network.

## Supplementary Information

**Additional file 1: Appendix 1 and 2.** Patient conversation templates.

**Additional file 2: Appendix 3.** GRIPP-2 SF.

## Data Availability

Videos and a case study of this CEI activity can be found on the GSU webpages.

## Abbreviations

CEI: Community Engagement and Involvement
GSU: Global Health Research Unit on Global Surgery (Global Surgery Unit)
HIC: High-income country
LMIC: Low- and Middle-Income Country
NIHR: National Institute for Health Research
PAG: Public Advisory Group
PPI: Patient and Public Involvement (CEI in UK context)
RCTs: Randomised Controlled Trials
TIGER: Task shifting inguinal hernia repair
WHO: World Health Organisation
