# Supplementary material for: Community engagement and involvement in Ghana: conversations with community stakeholders to inform surgical research
Source: Res Involv Engagem. 2021 Jul 5;7:50. doi: 10.1186/s40900-021-00270-5 (PMC8256583; doi:10.1186/s40900-021-00270-5)
Supplement: Supplementary file 1 — Additional file 1: Appendix 1 and 2. Patient conversation templates. [file 40900_2021_270_MOESM1_ESM.zip › Questioannaire 1.docxR3.pdf]

Design and acceptability of TIGeR study

The community angle

Questions for patient contributors living with hernia

Introduction of team
Hello, my name is ___. I work for the Global Surgery Unit in England. We want to ensure that our studies on certain conditions or surgical procedures are informed by people who live with these conditions and are likely to need surgery or have had surgery at some point. This helps us to ensure that our research is relevant and helps people who are affected by it.

Today, I am here to talk to you about a study on hernia surgery, called TIGeR, one of our studies designed by Prof Stephen Tabiri and his team here in Ghana. Here, we want to increase access to safe and affordable inguinal hernia repair in Ghana. We look into whether trained non-surgeon physicians can repair hernias as well as surgeons can.
These non-surgeon physicians will be trained by surgeons and supervised to ensure that they are qualified to then take on the procedure themselves.

This is about a condition you live with on a daily basis and your answers can really help us make a difference.

1. **Introduction of patient contributor**

To start, I would like to find out a little bit about you and your experiences of living with a hernia.

Name (First name sufficient):

Age:

Where do you live (Distance from hospital):

(scale, eg village, town, city, rural, urban)

Patient contributor's condition

Can you tell us about your hernia and its impact on your current everyday life?

Capture:

How long have you been living with a hernia?

What are your symptoms?

Does your condition stop you from living your everyday life?

Have you seen a doctor about it?

When/Why did you see a doctor (Symptoms got bad, pain etc) – How long after noticing the hernia did you see a doctor and did you treat it yourself first?

OR Why have you not seen a doctor/ seen a doctor sooner?

Was there anything stopping you from seeing a doctor when you noticed the hernia first? (Work, travel to hospital, prefer alternative medicine, self treatment)

Would you see a doctor sooner if they were to be local? Even if they might not be as qualified/not as much experience as the ones at the bigger hospitals?

Was surgery recommended and how long will you have to wait to have surgery? And do you know the reasons for waiting?

In between your initial appointment and your surgery, do you feel like you know how to look after your condition? Do you feel well taken care of?

Is your condition stopping you from going to work/living your life?

How do you feel about having surgery? What are your concerns? Do you trust your surgeon? Have you met them/would you like to meet them?

Hospitals and travel

Where did you see a doctor (District, main hospital, local)? Was this the most convenient for you or what would you prefer? What is the nearest hospital to you?

Have you been to a bigger hospital? What do you think are the main challenges of district hospitals (Resources, staff, equipment etc)?

Where are you having surgery? Is this your decision or your doctors and would you prefer it differently? Local or travel? Why?

How do you feel about visiting the hospital and why?

How long did your initial appointment take? And how long did the wait/travel take?

Were there any complications you encountered traveling to the hospital? And how could those be avoided?

What would have helped you to get from home to hospital more quickly (Is there a paved road to allow vehicles to come to your home? From your home are you able to access taxi services? Are they easy to get hold of? How much does a taxi cost? Do you, anyone you know, or anyone in your community own a motorcycle or car? In an emergency can these be used for transportation to hospital?)?

How much did it cost you to travel to hospital?

Did you miss a day at work and was this causing any problems?

How long did the actual appointment take?

Would you say it was an overall comfortable/uncomfortable visit? Why?

If uncomfortable, why and how can this be improved?

At your appointment, did you understand everything your doctor told you? Do you know much about your condition?

Did you mind who was seeing you as long as they were medically trained?

What is most important to you when you are at the hospital or be seen by a doctor? Care, hygiene, being seen fast, who sees you?

Would you rather have pain management and live with your hernia or have surgery?

Communication with doctors

How can you reach your doctor other than traveling to the hospital?

Do they ever travel to you? Would this make a difference?

Would you like to find out more about how to stay healthy? Or hernia specific information?

TIGeR

When hearing about TIGeR, was there anything that came to your mind immediately that you felt might be problematic or you have questions about? That you were not entirely happy with?

What do you perceive are the main challenges for people living with hernias?

Or people that are ill generally in your community? Anything surgery related? What do you feel are the main challenges to care in your community?

How relevant do you think this study is to you as a patient and your community? Will this make a difference to people living with hernias?
