# Supplementary material for: Community engagement and involvement in Ghana: conversations with community stakeholders to inform surgical research
Source: Res Involv Engagem. 2021 Jul 5;7:50. doi: 10.1186/s40900-021-00270-5 (PMC8256583; doi:10.1186/s40900-021-00270-5)
Supplement: Supplementary file 1 — Additional file 1: Appendix 1 and 2. Patient conversation templates. [file 40900_2021_270_MOESM1_ESM.zip › Questionnaire 2.docx (1)R3.pdf]

Design and acceptability of TIGeR study

The community angle

Questions for patient contributors who have had surgery

Introduction

Hello, my name is ____. I work for the Global Surgery Unit in England. To ensure that our studies on certain conditions or surgical procedures are informed by people who live with these conditions. This helps us to ensure that our research is relevant and helps people who are affected by it.

Today, I am here to talk to you about a study on hernia surgery, called TIGeR, one of our studies designed by Prof Stephen Tabiri and his team. Here, we want to increase access to safe and affordable inguinal hernia repair in Ghana. We look into if trained non-surgeon physicians can repair hernias with mesh as well as surgeons can.
These non-surgeon physicians will be trained by surgeons and supervised to ensure that they are qualified to then take on the procedure themselves.

This is about a condition you lived with on a daily basis and your answers can really help us make a difference.

Introduction of patient contributor

To start, I would like to find out a little bit about you and your experiences of living with a hernia and undergoing surgery for it.

Name (First name sufficient):

Age:

Where do you live? (Distance from hospital)

(scale, eg village, town, city, rural, urban)

Surgery - Capture:

How long had you been living with a hernia before having surgery?

What were your symptoms that led to you seeing a doctor? Did your condition stop you from living your everyday life?

Did you try pain management or alternative treatment before? Why?

How long did it take between your initial appointment and surgery? And do you know why it took this amount of time?

Was there anything stopping you from seeing a doctor when you noticed the hernia first? (Work, travel to hospital, prefer alternative medicine, self treatment)

Where did you see a doctor? Did you travel far to the next hospital or did you go to a local one or did the doctor come to you? Would you have seen a doctor sooner if they were to be local? Even if they might not be as qualified/not as much experience as the ones at the bigger hospitals?

In between your initial appointment and your surgery, did you feel like you knew how to look after your condition? Do you feel well taken care of?

How did you feel about having surgery? What were your concerns? Did you trust your surgeon? Did you meet the surgeon before your surgery?

Hospitals and travel

Where did you see a doctor (District, main hospital, local)? Was this the most convenient for you or what would you prefer? What is the nearest hospital to you?

Have you been to a bigger hospital? What do you think are the main challenges of district hospitals (Resources, staff, equipment etc)?

Where were you having surgery? Was this your decision or your doctors and would you prefer it differently? Local or travel? Why?

How did you feel about your surgery? Nervous, concerned?

What were the main concerns for you? Hygiene, surgery generally, missing work etc

And what is most important to you at hospital visit?

Can you tell me about the visit and the procedure?

How did you get to the hospital and how did you get bac home?

Were there any complications you encountered traveling to the hospital? And how could those be avoided?

What would have helped you to get from home to hospital more quickly (Is there a sufficient road to allow vehicles to come to your home? From your home are you able to access taxi services? Are they easy to get hold of? How much does a taxi cost? Do you, anyone you know, or anyone in your community own a motorcycle or car? In an emergency can these be used for transportation to hospital?)?

How much did it cost you to travel to hospital?

Did you miss a day at work and was this causing any problems?

How long did the actual surgery take?

Would you say it was an overall comfortable/uncomfortable visit? Why? Did you feel well taken care of? Why? And do you think this would have been different at a different hospital and why?

If uncomfortable, why and how can this be improved?

At your appointment, did you understand everything your doctor told you? Do you know much about your condition?

Did you ever wonder what your doctor's qualifications were and how many surgeries etc they had done before? Or were you just bothered about having it done as quickly as possible?

What is most important to you when you are at the hospital or be seen by a doctor? Care, hygiene, being seen fast, who sees you?

Would you rather have pain management and live with your hernia or have surgery?

Were you overall happy with the procedure?

Post- surgery

Did you go straight home after your surgery?

Tell us about those couple of days after your surgery? How much pain did you have, was there someone taking care of you? Did you know how to tend after your wound? Had you been given medicine?

When did you go back to work after your surgery and were you in a lot of pain still?

Did you have to go back into the hospital before your follow up because you had problems?

Follow up

Did you have to come back in for a follow up?

Was this a problem at all? Did you make the appointment?

Did you understand why the follow up was needed?

Did you get anything out of the follow up? Did you feel reassured afterwards that your wound had healed?

Did you lose out on payment at work?

How could this have been easier for you?

Communication with doctors

How can you reach your doctor other than traveling to the hospital?

Do they ever travel to you? Would this make a difference?

Would you like to find out more about how to stay healthy? Or hernia specific information?

TIGeR

When hearing about TIGeR, was there anything that came to your mind immediately that you felt might be problematic or you have questions about? That you were not entirely happy with? Do you think this study would have made a difference to you? Do you want to add anything we have missed?

What do you perceive are the main challenges for people living with hernias?

Or people that are ill generally in your community? Anything surgery related? What do you feel are the main challenges to care in your community?

How relevant do you think this study is to you as a patient and your community? Will this make a difference to people living with hernia?
