# Supplementary material for: Community engagement and involvement in Ghana: conversations with community stakeholders to inform surgical research
Source: Res Involv Engagem. 2021 Jul 5;7:50. doi: 10.1186/s40900-021-00270-5 (PMC8256583; doi:10.1186/s40900-021-00270-5)
Supplement: Supplementary file 2 — Additional file 2: Appendix 3. GRIPP-2 SF. [file 40900_2021_270_MOESM2_ESM.docx]

| Aim of CEI in the study | To inform relevance of study and acceptability and feasibility of the intervention |
| --- | --- |
| Methods used for CEI in this study | - Public advisory group discussions with UK-based members from LMICs: Reviewing of trial documents and CEI documents (focus on patient facing material)  - Patient consultations in Ghana: 1:1 semi-structured/guided conversations with translators (guidance questionnaires attached)  - Engagement activities with Chiefs and community leaders: Discussing burden of hernia repairs on communities and relevance of TIGER trial |
| CEI results/outcomes | - Amendment of study protocol (Inclusion of women)  - Confirming relevance, feasibility and acceptibility of study  - Supporting ethics application  - Establishing relationships with community leaders  - Dissemination pathways explored and what kind of information relevant to patients (Information on surgery and the risks, and hernias, their causes and the treatment options)  - Lessons learned and top tips for future CEI projects in LMICs  - Ghana research team realising importance and benefits of CEI and wanting to implement this in future studies |
| Reflections | - Patients worked well with us and were happy to contribute (Local team had concerns about their level of knowledge regarding research beforehand) – however, introduction of the related study and specifically the reason for the conversations had to be communicated in the right way in order to ensure that answers were based on patients’ actual opinions and thoughts and were therefore truthful and impactful (CEI v research)  - Translator has to be professional (not just a relative of patient), as it is crucial for them to translate exactly what has been said and have certain confidence in helping facilitate the conversation between GSU teams and patient contributor  - Local team’s understanding of CEI needs to be good – training needed before activities  - Roles of members of team need to be clear  - Flexible approach needed, things in LMICs may take longer or are handled differently  - Local team needs to lead on pathways to reach out to communities  - CEI activities can and should be implemented at a more sustainable, higher level at GSU now, eg community stakeholders as part of trial management groups and utilise established networks for dissemination of findings |

GRIPP-2 SF – CEI with TIGER
